# A new genus and three new species of noctuid moths from western United States of America and Mexico (Lepidoptera, Noctuidae, Noctuinae, Eriopygini)

**DOI:** 10.3897/zookeys.788.26068

**Published:** 2018-10-08

**Authors:** Lars G. Crabo

**Affiliations:** 1 724 14th Street, Bellingham, Washington 98225, USA Washington State University Bellingham, Pullman, Washington United States of America; 2 Adjunct Faculty, Washington State University, Pullman, Washington, USA Washington State University Bellingham, Pullman, Washington United States of America

**Keywords:** DNA barcode, Durango, Nuevo Léon, Rocky Mountains, Sierra Madre Occidental, Sierra Madre Oriental

## Abstract

The genus *Rhabdorthodes***gen. n.** is described for three previously unnamed noctuid moths from the mountains of south-western United States and Mexico. It is assigned to subfamily Noctuinae, tribe Eriopygini. *Rhabdorthodespattersoni***sp. n.** from the United States and *Rhabdorthodesdurango***sp. n.** and *Rhabdorthodespetersoni***sp. n.** from Mexico are described. These moths are small, dull gray brown, and lack highly diagnostic wing markings, but are distinctive structurally. The adults and genitalia of both sexes are illustrated and distribution maps are presented. Two species eponyms honor persons who have facilitated the study and enjoyment of moths in North America by creating moth-specific websites.

## Introduction

Lafontaine and Schmidt (2010) produced the first new major check list of Canadian and United States Noctuoidea since Hodges et al. (1983). Discoveries of novel Erebidae and Noctuidae have continued unabated since then, with over 40 newly described species added to the fauna in subsequent updates (Lafontaine and Schmidt 2011, 2013, 2015). Herein, yet another new species is named, a widespread nondescript noctuid moth from the Rocky Mountain region of the United States. In contrast to the drab superficial appearance of this moth, reproductive structural features of both sexes are far from ordinary - especially the male juxta and valves and female sternite A7–warranting a new genus for it. In males, a long rod extends posteriorly from the juxta and the distal claspers are massive and ornate. The most distinctive features of females are a fleshy frond like structure at the posterior ductus bursae and external sculpting of the lateral seventh sternite, the latter modification likely to receive the male claspers during coupling.

In addition to the new species from the United States, two unnamed moths from the mountains of Mexico belonging to this genus were discovered amongst unsorted material at the Canadian National Collection.

I honor two persons who have contributed to the study and enjoyment of moths on the Internet by naming moths after them. These tools make accessible to anyone with a computer what was previously the domain of experts with access to large collections and rare literature sources.

## Materials and methods

Wing pattern and genitalia structure terminology follow Lafontaine (2004). Forewing length is measured to the nearest half millimeter from base to apex, excluding the fringe. Genitalia are prepared using standard methods (Hardwick 1950, Lafontaine 2004). Detached abdomens are macerated in hot 10% potassium hydroxide for 20–40 minutes. Dissection is performed initially in water or a 30:70 ethanol-water mixture followed by hardening in isopropyl alcohol. Male vesicas and female bursae are inflated. Preparations are stained with orcein [Sigma Chemical Company, St. Louis, Missouri] and mounted in Euparal [Bioquip Products, Rancho Dominguez, California] under elevated cover glass on glass slides.

The 658 base pair “barcode” region of mitochondrial cox1 *mt* DNA was used to assess the taxonomic placement of the new genus. Legs from dried specimens submitted to the Barcodes of Life Data System (BOLD) at the University of Guelph (Ontario, Canada) were analyzed by standard DNA extraction, amplification, and sequencing protocols (Hebert et al. 2003). Barcodes were compared to pre-existing material at BOLD as implemented on the website (http://www.barcodinglife.org). The seven-unit BOLD Barcode Index Number (BIN) (Ratnasingham and Hebert 2013) is given for *Rhabdorthodespattersoni* Crabo.

Distribution maps were made using SimpleMappr (http://simplemappr.net).

Repository abbreviations:

**CNC** Canadian National Collection of Insects, Arachnids, and Nematodes, Ottawa, Ontario, Canada

**LGC** Lars Crabo Collection, Bellingham, Washington, USA

**TM** Tomas Mustelin Collection, Seattle, Washington, USA

## Taxonomy

### Rhabdorthodes gen. n.

Taxon classificationAnimaliaLepidopteraNoctuidae

http://zoobank.org/4AFE2E20-0A73-42D0-B79D-FA7CB559F05D

#### Type species.

*Rhabdorthodespattersoni* Crabo.

#### Gender.

Masculine.

#### Diagnosis.

*Rhabdorthodes* is a distinctive genus with characteristic male and female genitalia. The adults are small to medium-sized, forewing length 13–15.5 mm, and nondescript superficially, with even gray-brown to brown forewings with limited dark markings. The eyes have interfacetal setae. Males have a long rod like extension of the right side of the dorsal juxta (Figs 7–9) that is unknown in any other North American genus. The valves are also distinctive, with massive slightly bilaterally asymmetrical claspers with horn- and molar like processes. Females have diagnostic features of the bursa and segment A7. The posterior ductus bursae is expanded into an amorphous fleshy externally frondlike sac (Figs 10–12). Externally, sternite A7 is broad, sclerotized strongly, with a broad sculpted depression on the ventrolateral surface to each side of midline (Figs 13–15). This is most apparent in intact specimens and is observed easily under low magnification. The papillae anales are very thin and needlelike and the distal abdomen is telescopic with long intersegmental membranes.

The most closely related genus based on structure and barcodes is *Protorthodes* McDunnough, revised recently by Lafontaine et al. (2014). Males of *Protorthodes* lack the rodlike extension of the juxta that is found in *Rhabdorthodes* and have a normal-sized uncus. The valve of *Protorthodes* species differ from those of *Rhabdorthodes* in having a long thin ampulla of the clasper arising from the mesial surface of the valve rather than a stout curved one at the dorsal margin, and lack massive enlargement of the distal clasper with a large ventral extension. Females of *Protorthodes* lack or have only a small fleshy components of the anterior ductus bursae, well developed in *Rhabdorthodes*, and lack sculpting of the ventral seventh sternite.

The barcode of *Rhabdorthodespattersoni* is closest to *Protorthodes*, differing from barcodes of species in this genus by at least 3.5%. The two Mexican species of *Rhabdorthodes* have not been barcoded.

#### Description.

**Adults.** Males and females similar in habitus. *Head.* Antenna biserrate, rami densely setose, total width 3–4 × shaft, anterior rami slightly longer than posterior rami (male); beadlike, biciliate (female); dorsum with small scales. Eye normal size, interfacetal setae long, curved apically. Labial palpus reaching dorsal margin of eye; sides of first two segments with short strap like scales, anterior first segment with medium-length simple and long hairl-ike scales, anterior second segment scales similar, shorter; apical segment 0.2 × second segment, scales very short. Haustellum normal. Frons unmodified, scales simple, medium length; dorsal head scales long, thin, spatulate, forked.

*Thorax.* Dorsal vestiture dense, long, thin, spatulate and forked scales, medium to dark gray brown; weak mesial tuft on anterior metathorax. Venter scales hair like, dense, dull brown. *Legs*: Tibiae without claws or other modifications, dark gray brown with scattered off-white scales; tarsi except apical segment with three rows of spine like setae, segments dark brown, ringed distally in off-white. *Wings*: Forewing: Length 13.0–14.0 mm (males), 13.5–15.5 mm (females), length ~ 2 × width; outer margin smoothly convex, strongest near anal angle; dorsal scales short, straplike, uniform medium to dark gray brown or brown; costa with 6 light spots on basal, antemedial, postmedial line origins and 3 spaced evenly between postmedial line and apex; lines and stigmata except subterminal line black; lines double, filling pale; subterminal line pale gray or luteous, preceding shade dark brown or black; fringe ground color. Hindwing: Outer margin slightly concave M1–M3; dorsum gray brown, lightest basally; veins and terminal line dark; fringe lighter than ground.

*Abdomen.* Male unmodified. Female sternite A7 (Figs 13–15) sclerotized, thickened posteriorly; posterior margin pointed bluntly in midline, concave to each side of midline; ventrolateral surface sculpted with broad central concavity with lateral raised flange (two species) or deep transverse cleft (*R.petersoni*). *Male genitalia*: Uncus weak, length 0.5–1 × juxta height, shorter and thinner than juxta extension, curved evenly, tapered to thin point, distal undersurface with short thin hair like setae. Juxta base hourglass shaped, elongate, height 2.2–3.3 × width; dorsum asymmetric: left lobe small, flat; right with long stout tapered rod, length 1.2–1.5 × juxta height, projecting posteriorly with slight curve ventrad and leftward. Valve length 4.3–6.0 × width, weak distal to clasper; sacculus 0.5–0.6 × valve length and 0.75–0.8 × valve width, smooth; ventral distal clasper distal to ampulla heavily sclerotized, massive, 1.0–1.2 × valve width, dorsal and ventral toothlike and hornlike processes extending beyond valve margins (dorsal extension reduced in one species); ampulla origin from bulging clasper at dorsal valve, stout, hook-shaped, base oriented 30–45° basad or perpendicular to valve, then curved distad 90–180°; digitus thin, membranous, directed distad from origin near ventral valve; cucullus weak, rounded, barely wider than “neck,” covered densely by short thin hairlike setae, lacking corona. Phallus tubular, narrow, length ~ 10 × width. Vesica membranous, length ~ 1.5 × and width 2.5 × phallus, base bent 90° left, mid-section coiled 360° counter-clockwise, distal segment bent 45° cephalad to end right or ventral to phallus; small foot-shaped basal diverticulum; no cornuti. *Female genitalia*: Papilla analis length 6–8 × width, thin, pointed, covered sparsely with short thin hairs on lateral surface and densely with very short thin hairs on medial tip; posterior intersegmental membranes long, eversible. Segment A8 length 1.25–1.50 × width, with sparse short thin setae; posterior apophysis 2.1–2.4 × segment A8 length; anterior apophysis 0.4 × posterior apophysis. Ostium bursae sclerotized lightly with ventral short median cleft or leathery, lacking cleft. Ductus bursae 1.5 × segment A8 length; posterior third sclerotized dorsally, expanded to fleshy frondlike structure filling most of ventral segment A7; middle third sclerotized, tubular; anterior third membranous, tubular. Corpus bursae bisaccate, corpus bursae and appendix bursae similar size; corpus bursae membranous, ovoid, length 2 × width, lacking signa; appendix bursae origin from left posterior corpus bursae, curved 270° anterior, leftward, and posterior to end to left of mid-ductus bursae; ductus seminalis at apex.

#### Etymology.

The name is derived from the Greek *rhabdos*, meaning rod, and *Orthodes*, a genus of moths in the tribe Eriopygini. It refers to the long extension of the male juxta.

#### Distribution and ecology.

*Rhabdorthodes* species occur in the mountains of western United States and Mexico from southern Idaho and southern Wyoming in the United States to Nuevo Léon and Durango in Mexico. Adults fly in the summer during June and July. All three species in the genus have been collected in montane forests at mid- to high elevations between 1600 and 3150 meters. The early stages are unknown for all species.

#### Discussion.

Assignment of this genus to subfamily Noctuinae tribe Eriopygini is based on the presence of hairy eyes, similarity of the adults to species of other genera in this tribe, and the association of the barcode of *R.pattersoni* sp. n. with those of *Protorthodes* McDunnough on neighbor-joining trees. Although almost certainly correct, this is provisional until the early stages are discovered. The main morphologic difference between tribes Hadenini and Eriopygini is in the mandible and spinneret of the larva (Fibiger and Lafontaine 2005), currently unknown for *Rhabdorthodes*. This classification is supported by the presence of a long coiled vesica in males of *Rhabdorthodes*, since this is a typical feature of many species in the tribe Eriopygini (Fibiger and Lafontaine op. cit.).

The functions of the unique sexual characters of *Rhabdorthodes* can only be surmised. In males, the long rodlike extension of the juxta, diminutive uncus, and the massive sculpted ampulla of the clasper with molar- and hornlike processes are unlike any other in the Eriopygini. In the female, the fleshy enlargement of the posterior ductus bursae and the sculpted lateral segments A7 are similarly unusual. The needlelike ovipositor and telescopic distal abdomen is also distinctive, although similar modifications are known in other taxa.

The fleshy posterior ductus bursae appears gland like and might have a secretory function. It could potentially have a mechanical function as well, receiving the long juxta during copulation. Even if coupling does not occur in this fashion, the male rod must somehow engage the female. The weak uncus suggests that the rod might have supplanted all or part of its function. Similarly, the massive claspers of the distal male valve probably engage the concave pits on the posterior female abdomen. The latter modifications are analogous to those of the noctuid genus *Spaelotis* Boisduval (Noctuinae, Noctuini)–illustrated in Lafontaine (1998: 73)–in which females have species-specific pits on the ventral posterior abdomen that likely receive the ampullae of the male claspers. The needlelike papillae anales and long eversible posterior abdomen of *Rhabdorthodes* suggest that females lay eggs deep within a specific plant structure, less likely deep in soil.

### Key to *Rhabdorthodes* adults

**Table:** 

1	Male	**2**
–	Female	**4**
2	Clasper distad of ampulla with similar sized dorsal and ventral processes, ventral process triangular (Figure 7); United States	*** R. pattersoni ***
–	Clasper distad of ampulla asymmetrical, ventral process long, curved toward valve apex (Figs 8, 9); Mexico	**3**
3	Clasper distad of ampulla two pronged, with molarlike dorsal and curved ventral processes (Figure 8); Sierra Madre Occidental	*** R. durango ***
–	Clasper distad of ampulla with single curved ventral process (Figure 9); Sierra Madre Oriental	*** R. petersoni ***
4	Concave lateral part of sternite A7 with deep transverse sulcus (Figure 15); Sierra Madre Oriental, Mexico	*** R. petersoni ***
–	Lateral segment A7 concave without transverse sulcus (Figs 13, 14); United States or Sierra Madre Occidental, Mexico	**5**
5	Concave part of sternite A7 shallow, lateral margin quadrate with weakly raised rim (Figure 13); United States	*** R. pattersoni ***
–	Concave part of sternite A7 deeper, lateral margin rounded with lateral flange resembling the helix of a human ear (Figure 14); Sierra Madre Occidental, Mexico	*** R. durango ***

### Species accounts

#### Rhabdorthodes pattersoni sp. n.

Taxon classificationAnimaliaLepidopteraNoctuidae

http://zoobank.org/DCF3F28F-22BA-473C-B9AC-16A03F32F5CD

Figs 1–4 , 7 , 10 , 13 , 16

##### Type locality.

USA: Colorado: Clear Creek County: Doolittle Ranch, Mt. Evans, 2987 m.

##### Type material.

**Holotype, male.** USA: Colorado: [Clear Creek County]: Doolittle Ranch, Mt. Evans, 9,800’ [2987 m], 10 VIII 1961, E. W. Rockburne. / Specimen ID CNCLEP 00140423. CNC. **Paratypes.** 49 m, 5 f. **USA**: **Arizona**: Apache County: Alpine, 3–5 mi [4.8–8.0 km] SE, 16 VI 1967 (1 m); Greer, White Mts., 8500’ [2591 m], 6 VIII 1962, E. & I. Munroe, black light (1 m); same collection label / Genitalia slide # 11,732 male (1 m); [Greenlee County], Hannagan Meadow, 13 VI 1967, R. F. Sternitzky / Genitalia CNC slide # 17406 female (1 f); same collection label / [CNC] Slide No. 11,734 (1 m); **Colorado**: Archuleta County: [north of Pagosa Springs], 8[000]’ [2438 m.], 15 VI 2004, Vargo leg. / Specimen ID CNCLEP 00120092 (1 m); same collection label / Specimen ID CNCLEP 00120092 (1 f); [Boulder County]: Boulder, Silver Saddle Motel, 5500’ [1676 m], 5 VI 1961, M. R. MacKay (1 m); [Clear Creek County]: Doolittle Ranch, Mt. Evans, 9800’ [2987 m], 16 VII 1961, E. W. Rockburne / Genitalia CNC slide # 15894 male (1 m); same collection label, 30 VII 1961 (1 m); same collection label / [CNC] Slide No. 10,766 female (1 f); same locality & collector, 31 VII 1961 (4 m); same collection label / [CNC] Slide No. 10,764 male (1 m); same locality & collector, 1 VIII 1961 (8 m); same locality and collector, 2 VIII 1961 (6 m); same collection label / Genitalia CNC slide # 15893 male (1 m); same locality & collector, 3 VIII 1961 (1 m); same locality & collector, 5 VIII 1961 (1 m); same locality & collector, 6 VIII 1961 (1 m); same locality & collector, 6 VIII 1961 (1 m); same locality & collector, 8 VIII 1961 (1 m); Teller County: Florissant, 3.5 mi [5.6 km] SW, 38.904° -105.323°, 8–9 VII 2016, 2660 m, L. G. Crabo & G. Morrell leg. (4 m); **Idaho**: Bear Lake County: Georgetown Cyn., 42.524° -111.263°, 2100 m, 4 VII 2016, L. G. Crabo & G. Morrel leg. / Specimen ID CNCLEP00140350 / Barcode of Life Project, Leg removed, DNA extracted (1 m); **New Mexico**: Colfax County: Cimarron Canyon, Sangre de Cristo Mts., 7900’ [2408 m], 6 VII 1982, black light, E. & I. Munroe (1 m); same collection label / [CNC] slide No. 11733 male (1 m); same locality & collector, 8 VII 1962 (1 m); same locality & collector, 11 VII 1962 (1 m); Lincoln County: Capitan Mts., Capital Ridge, radio towers, summit, 10,000’ [3048 m], 3 VII 1982, RWH [Ronald H. Hodges] (2 m); same locality & collector, 10 VII 1982 / Genitalia slide # 17407 female (1 f); Otero County, High Rolls, Karr Cyn., 32.898° -105.813°, 2400 m, 9 VI 2016, L. G. Crabo leg. (1 f); Sandoval County: San Jose Ca[illegible]., 4 mi [6.4 km] E. Regina, Jemez Mts., NW slope, 8500’ [2591 m], 26 June, 1983, UV, RWH [Richard W. Holland] (1 m); **Utah**: [Iron County]: Cedar City, 11 mi [17.7 km] SE, 8300’ [2530 m], 29 VIII 1965, D. F. Hardwick (1 m); Sanpete County: Ephraim, 8 mi [12.9 km] E, 39.317°–[39.]337° -111.448°–[111.]470°, 10000’ [3048 m], 21 VII 2006, L. G. Crabo leg. / Specimen ID CNCLEP00140348/Barcode of Life Project, Leg removed, DNA extracted (1 m); same collection label / Specimen ID CNCLEP00140349 / Barcode of Life Project, Leg removed, DNA extracted (1m); **Wyoming**: Albany County: T13N R77W, Section 4, 1.5 mi (2.6 km) NW of Woods Landing, Fox Creek, el. 7,600 ft (2316 m), 21 VI 1997, black light trap, J. S. Nordin leg. (1 m): CNC, LGC, TM.

**Figures 1–6.**
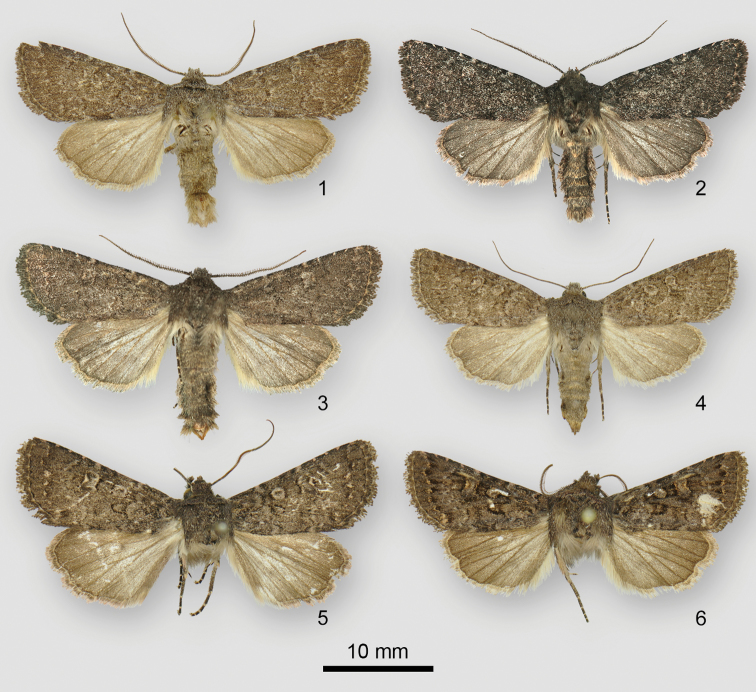
*Rhabdorthodes* adults **1***R.pattersoni*, holotype male, USA, Colorado, Clear Creek County, Doolittle Ranch, Mt. Evans **2***R.pattersoni*, paratype male, USA, Colorado, Teller County, Florissant, 5.6 km SW **3***R.pattersoni*, paratype male, USA, Utah, Sanpete County, Ephraim, 12.9 km E **4***R.pattersoni*, paratype female, USA, Colorado, Archuleta County, Pagosa Springs **5***R.durango*, holotype male, Mexico, Durango, El Salto **6***R.petersoni*, holotype male, Mexico, Nuevo Léon, Cerro Potosi.

##### Diagnosis.

*Rhabdorthodespattersoni* is the most drab and poorly patterned of the three *Rhabdorthodes* species, appearing dull dark brown with faint markings. The forewing subterminal line of *R.pattersoni* is faint pale gray, whereas those of the Mexican species are more prominent, luteous preceded by dark wedge-shaped spots. *Rhabdorthodespattersoni* is the only species in the genus that is known to occur in the United States.

**Figures 7–9.**
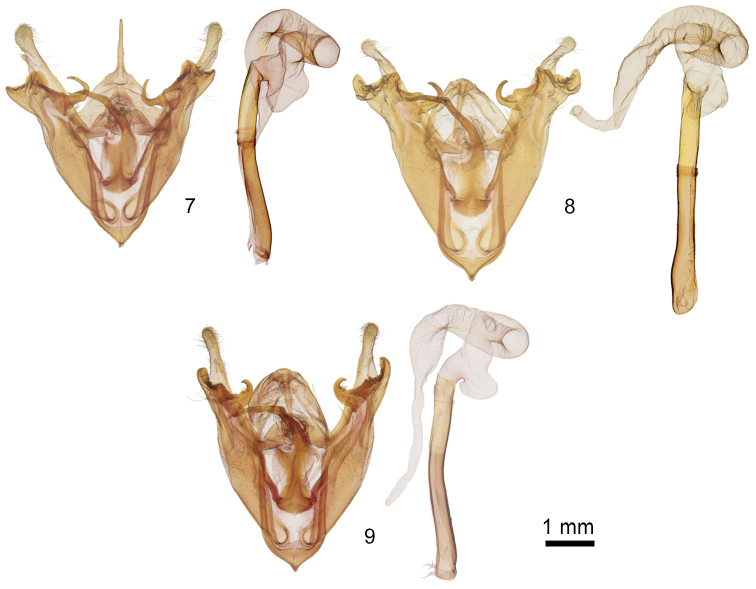
*Rhabdorthodes* male genitalia, valves and phalluses with everted vesicas **7***R.pattersoni***8***R.durango***9***R.petersoni*.

Structurally, males of *R.pattersoni* are distinguished from the Mexican species by the smaller ventral process of the distal clasper (Figure 7), relatively short and triangular. In both other species this process is longer and curves toward the apex of the valve.

The shape of sternite A7 is shallowly concave with a quadrate lateral margin (Fig. 13). Those of the two Mexican species are more complex, with distinct lateral flanges, deeper pits, and more strongly concave edges (Figs 14, 15).

The Barcode Index Number (BIN) of *R.pattersoni* from Colorado and Utah (*n* = 5) is BOLD:ADH0770.

This moth resembles several nondescript brown species of Eriopygini – such as species of *Homorthodes* McDunnough and *Protorthodes* – but is probably most similar superficially to “*Orthosia*” *noverca* (Grote), a common widespread western North American moth that lacks a satisfactory generic placement. Most “*O.*” *noverca* have thicker and more prominent black forewing pattern elements than those of *R.pattersoni*. *Rhabdorthodes* can be identified without dissection by observing the ends of the valves for the chunky claspers in males and the sculpted seventh sternite in females. In addition, males of *Rhabdorthodes* have biserrate antennae, simple in look-alikes other than *Protorthodes*.

##### Description.

**Adults.***Head.* Male antenna total width 3 × shaft; dorsal scales dark gray-brown, scattered off-white on proximal ⅓. Labial palpus scales gray-brown, scattered off-white on second and third segments, increasing toward tip. Frons scales gray brown; dorsal head scales white tipped gray-brown.

*Thorax.* Dorsal scales long, white tipped gray-brown; appearing uniform dull gray-brown to dark brown. *Wings*: Forewing length 13.0–13.5 mm (males); 13.5 mm (females); scales gray-brown, scattered white tipped gray-brown, appearing uniform gray-brown to dark brown; costa spots luteous gray; basal, antemedial, and postmedial lines double, black and dark gray-brown, filling ground and slightly lighter gray; basal line uneven, indistinct; antemedial line slightly irregular, pointed basad on veins; medial line slightly darker than ground, angled distad from mid costa to reniform stigma, thence basad to posterior margin; postmedial line indistinct, evident mostly as black inner part and pale gray filling, scalloped; subterminal line irregular off-white patches between veins, preceding shade faint, brown; terminal line thin, dark gray; fringe striped dark gray, base thin, pale; orbicular stigma round, often incomplete, black, lined with few pale-gray scales, center ground color; claviform stigma small, black, filled with ground color, or reduced to a spot; reniform stigma moderate size, weakly figure-8 shaped, open at posterior end, black, with adjacent luteous and pale-gray lining strongest at medial and lateral sides, center ground color. Hindwing: Dorsum gray, basal ½ paler; veins and faint discal spot dark; terminal line thin, black; fringe gray with copper luster, base thin, pale.

**Figures 10–12.**
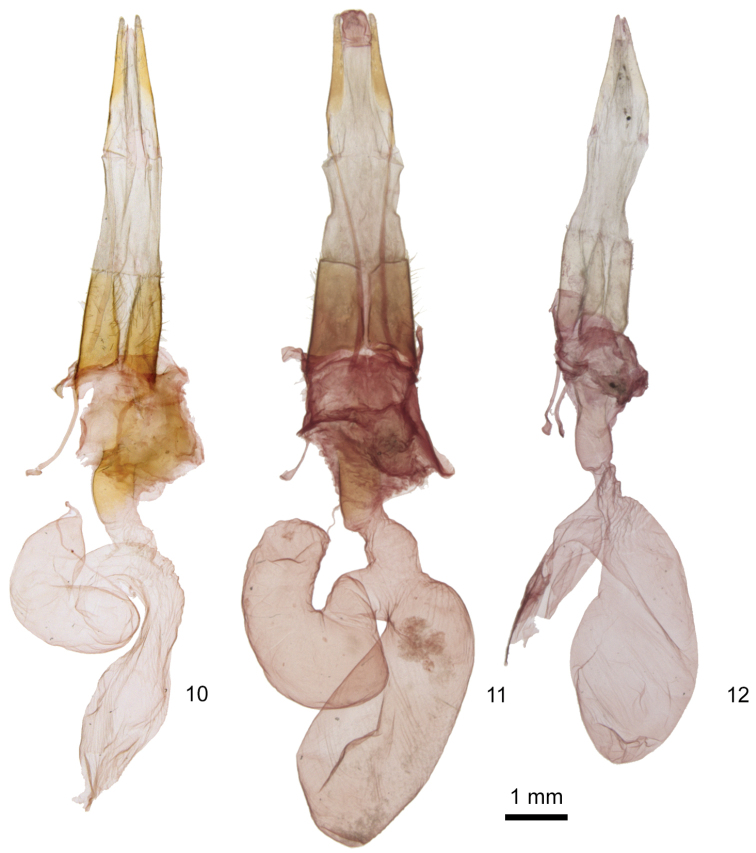
*Rhabdorthodes* female genitalia **10***R.pattersoni***11***R.durango***12***R.petersoni*.

**Figures 13–15.**
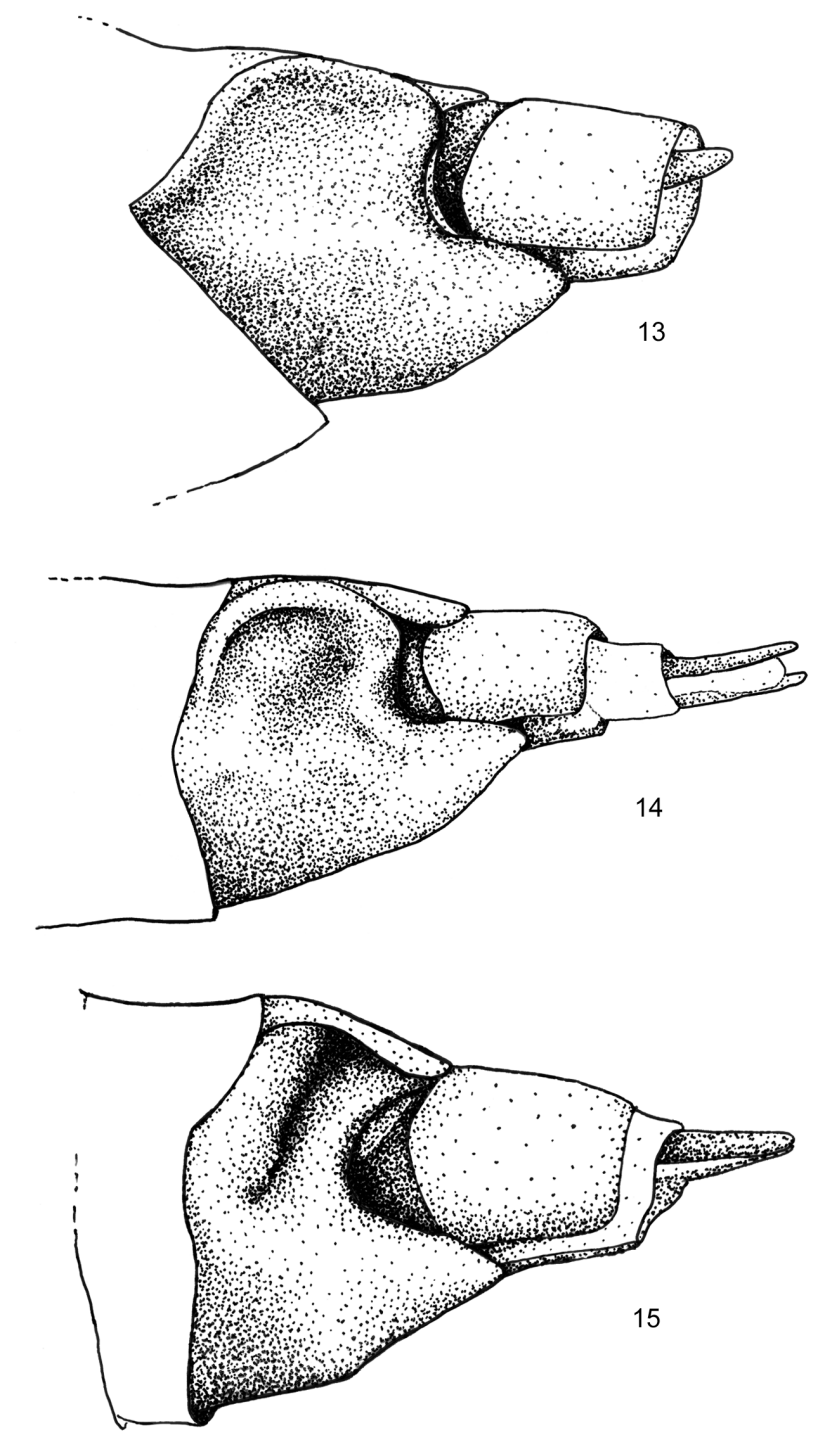
*Rhabdorthodes* females. Distal abdomen (scales removed), left ventrolateral aspect showing sculpted sternite A7. **13***R.pattersoni***14***R.durango***15***R.petersoni*.

*Abdomen. Male genitalia*: (Figure 7) Uncus length 0.9 × juxta. Juxta height 2 × width, rod length 1.2 × height. Valve length 5 × width, part distal to clasper curved slightly dorsad; cucullus weak, paddle shaped; sacculus length 0.5 × and width 0.75 × valve; distal clasper 1 × valve width, left slightly larger than right, thick, mesial surface with molar like ridges, dorsal distal margin expanded to broad rounded projection, ventral distal margin triangular with slight curve distad; ampulla directed 45° basad, scythe shaped with 120–180° curve distad; digitus small, thin. Phallus and vesica as in genus description. *Female genitalia* (Figs 10, 13): Sternite A7 lateral concavity shallow with quadrate raised lateral margin; posterior margin weakly concave. Papilla analis, segment A8, and bursa copulatrix as in genus description.

##### Etymology.

I am pleased to name this moth after Robert (Bob) Patterson of Bowie, Maryland in recognition of his contribution to the study and enjoyment of North American moths through his work on the Moth Photographers Group website (http://mothphotographersgroup.msstate.edu).

##### Distribution and ecology.

**Figure 16.**
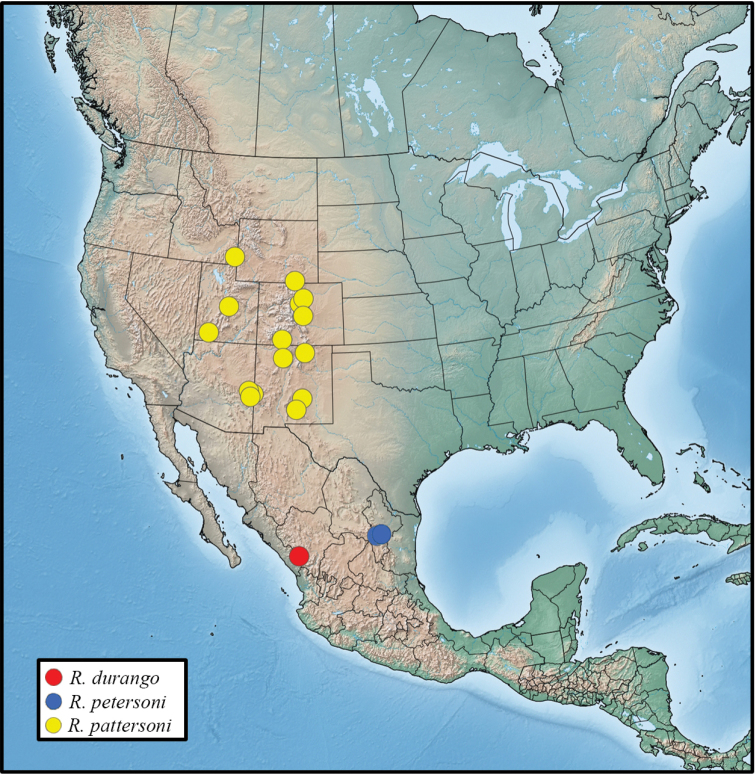
Distribution of examined material of *Rhabdorthodes* in North America. *R.pattersoni* (yellow), *R.durango* (red), *R.petersoni* (blue).

*Rhabdorthodespattersoni* occurs in the mountains of the American West (Figure 16). It has been collected from southeastern Idaho and south-central Wyoming in the north to southwestern Utah, eastern Arizona and south-central New Mexico in the south. Most records are from Colorado and New Mexico. It flies in mid- to high-elevation forest at elevations from 1600 to 3050 meters. Collection dates range from early June to early August. The early stages are unknown.

##### Remarks.

This species is moderately common in collections. Specimens are often mixed in with other brown species in the tribe Eriopygini, often “*Orthodes*” *noverca* and “*Orthodes*” *obscura* (Smith).

#### Rhabdorthodes durango sp. n.

Taxon classificationAnimaliaLepidopteraNoctuidae

http://zoobank.org/6622B61A-035E-4DD0-BEFA-99DD5FAA5761

Figs 5 , 8 , 11 , 14 , 16

##### Type locality.

Mexico: Durango: 16 km west of El Salto, 2743 m.

##### Type material.

**Holotype, male.** Mexico: D[uran]go: 10 mi. [16 km] W El Salto, 9000’ [2743 m], 8 VIII 1964, J. E. H. Martin. / Specimen ID CNCLEP 00140425 / Genitalia CNC slide # 17405 male. CNC. **Paratype**. 1 male, 1 female. Same locality as holotype, 9 VIII 1964, W. C. McGuffin / Genitalia CNC slide 15892 male (1 male); same label data as last / Genitalia CNC slide 17437 (1 f). CNC.

##### Diagnosis.

This species and *R.petersoni* sp. n. are closely similar and cannot be distinguished reliably by superficial appearance. Both are more strongly patterned and have more luteous filling of lines and stigmata on the forewing than *R.pattersoni*. In practice, location provides a convenient diagnostic character since it is likely that all three species are allopatric. *Rhabdorthodesdurango* sp. n. is the only species that is known from the Sierra Madre Occidental, Mexico.

Structurally, males of *R.durango* have the largest and most complex distal clasper in the genus, with a blunt curved ventral process that extends ventral to the valve and a molar like mesial process that projects over the distal valve. By comparison, the distal clasper of other species is much smaller (*R.pattersoni*) or lacks the broad mesial process (*R.petersoni*).

Females of *R.durango* can be identified by the shape of the concave ventrolateral sternite A7 (Figure 14). The concavity is similar to that of *R.pattersoni* in that it lacks a transverse sulcus, but the lateral edge of *R.durango* is rounded rather than quadrate. The lateral edge is shaped like the pinna of a human ear.

##### Description.

**Adults.***Head.* Male antenna total width 3 × shaft; dorsal scales dark gray-brown, occasional off-white on basal third. Labial palpus scales dark gray-brown, occasional off-white. Frons brown; dorsal head scales long, thin, mostly forked, dark gray-brown with pale bases and tips.

*Thorax.* Dorsum scales similar to dorsal head, appearing uniform dark gray-brown; venter darker. *Legs*: Scales dark gray brown, few off-white; tarsal segments darker brown ringed distally off-white. *Wings*: Forewing: Length 14 mm (male); 15.5 mm (female); dorsal scales nearly uniform dark gray-brown, costa black-brown; costa spots dark ochre-gray; short white veins segments: A1+A2 in proximal medial area, several in subterminal area; basal, antemedial, and postmedial lines double, black and dark-gray brown, filling paler brown-gray; basal line near wing base, indistinct; antemedial line slightly irregular, perpendicular to wing; medial line very faint, evident on anterior ⅓; postmedial line indistinct, scalloped weakly, evident on costa and posterior to reniform stigma; subterminal line ochreous off-white, irregular, patchy, strongest between branches of medial vein, preceded where strongest by ill-defined triangular black spots; terminal line thin, black, small pale spots on tips of veins; fringe ground color, edge slightly paler; claviform stigma small, black, pale filling like adjacent antemedial line; orbicular stigma nearly round, black, pale brown gray peripherally, ocellus black; reniform stigma moderately large, figure-8 shaped, black, lacking anterior and posterior outline in HT, posterior in PT, filling similar to orbicular medially, luteous laterally, ground color centrally. Hindwing: Dorsum dull medium fuscous, slightly paler on basal ½; veins and indistinct discal spot darker, faint; terminal line thin, black; fringe base dark gray, distal lighter ruddy gray.

*Abdomen. Male genitalia* (Figure 8): Uncus length 0.5 × juxta height. Juxta length 2.2 × width, rod length 1.5 × juxta. Valve length 4.3 × width, segment distal to clasper diminutive, curved slightly ventrad; cucullus very weak, rounded; sacculus length 0.6 × and width 0.8 × valve; distal clasper 1.2 × valve width, right slightly larger than left, thick, mesial surface with molar like ridges, dorsal margin expanded slightly at ampulla base, mesial lobe broadly triangular, oriented distad along valve axis, ventral process horn shaped, curved 90° distad; ampulla of clasper stout, directed 30° basad, distal ampulla hook shaped with 120–180° curve distad; digitus small, thin. Phallus and vesica as in genus description. *Female genitalia* (Figs 11, 14): Ventrolateral sternite A7 concave, deeper than in *R.pattersoni*, lateral margin rounded with raised flange similar to the dorsal helix of a human external ear; posterior margin thicker and more strongly convex than in *R.pattersoni*, less than in *R.petersoni*. Papilla analis, segment A8, and bursa copulatrix as in genus description; ostium bursae sclerotized with median cleft.

##### Etymology.

The species name refers to the type locality in the state of Durango, Mexico. It is a noun in the nominative singular in apposition to the generic name.

##### Distribution and ecology.

*Rhabdorthodesdurango* is known only from the type locality near El Salto in the Sierra Madre Occidental (Figure 16). The area is reported to be open pine-oak forest (D. Lafontaine, pers. Comm.). All three specimens were collected on consecutive days in early August. The early stages are unknown.

##### Remarks.

This species was found by J. Donald Lafontaine amongst unsorted Mexican material at the CNC. Although the type locality is nearly 950 km south of Arizona, it is conceivable that *R.durango* could occur on higher mountains in the Madrean Archipelago ecoprovince of south-eastern Arizona, the northernmost extension of the Sierra Madre Occidental. Many of the species collected in this CNC survey near El Salto occur in the mountains of southeastern Arizona (D. Lafontaine, pers. Comm.).

#### Rhabdorthodes petersoni sp. n.

Taxon classificationAnimaliaLepidopteraNoctuidae

http://zoobank.org/D5809F65-2F03-495F-AA72-037AABAD6498

Figs 6 , 9 , 12 , 15 , 16

##### Type locality.

Mexico: Nuevo Léon: Cerro Potosi, 3139 m.

##### Type material.

**Holotype, male.** Mexico: N.[uevo] L.[éon], Cerro Potosi, 10,300’ [3139 m], 15–16 VII 1963, H. & A. Howden. / Genitalia CNC slide No. 16495 / Specimen ID CNCLEP 00140417. CNC. **Paratype**. Female. Same data as holotype / Genitalia CNC slide 17438 female. CNC.

##### Diagnosis.

This moth is nearly identical to the other Mexican species, *R.durango*. Both Mexican species are more strongly patterned on the forewing than *R.pattersoni*, with stronger black markings and more vivid yellow filling of lines and stigmata. This yellow color is slightly more orange in *R.petersoni* than in *R.durango*. Definitive diagnosis requires examination of the genitalia, although the moths can most easily be identified by locality. *Rhabdorthodespetersoni* flies in the Sierra Madre Oriental and *R.durango* occurs in the Sierra Madre Occidental.

The base of the ampulla of the clasper of *R.petersoni* is oriented perpendicular to the dorsal valve, whereas those of the other two species are directed basad. The ventral process of the clasper is thinner and less strongly curved than in *R.durango*, and there is no significant dorsal process between the ampulla and ventral process. Together, the ampulla and ventral clasper process give the impression of an open lobster claw in *R.petersoni*.

The *R.petersoni* female is the only species in the genus that has a deep transverse sulcus across the ventrolateral part of sternite A7 (Figure 15).

##### Description.

**Adults.***Head.* Male antenna width 4 × shaft; dorsal scales dark gray brown, scattered off-white on basal ⅓. Labial palpus proximal segments scales gray brown, scattered off-white; distal segment pale tipped gray brown. Frons gray brown; dorsal head scales hair like gray brown, pale tipped forked gray brown.

*Thorax.* Scales similar to head, slightly paler posteriorly; appearing uniform gray-brown, weak pale collar edge. *Wings*: Forewing length: 13.0 mm (male), 13.5 mm (female). Dorsum purplish dark gray-brown, costa black-brown, costa spots dark ochre; basal, antemedial, and postmedial lines double, black and dark gray, filling paler brown-gray; basal line scalloped, incomplete; antemedial line irregular, pointed basad on veins, perpendicular to wing; medial line faint, dark, evident costa to medial reniform stigma; postmedial line indistinct, scalloped, black inner component and pale filling most evident; subterminal line sinuous, pale ochre, strongest between veins, preceded by dark shade forming black wedges opposite cell and in fold; terminal line intervenal dark gray triangles; fringe ground color, base pale, thin; claviform stigma small, black, filling pale, brassy; orbicular stigma nearly round, moderately large (fused to claviform stigma in PT), black, filling slightly paler than line filling, central ocellus weak; reniform stigma moderately large, weakly kidney shaped, black, filling orange ochre peripherally, gray brown centrally. Hindwing: Dorsum dull medium fuscous, slightly paler on basal ½; veins and indistinct discal spot barely darker; terminal line thin, black; fringe gray, base luteous, edge light gray.

*Male genitalia* (Figure 9): Uncus length 1 × juxta height. Juxta height 3.3 × width, rod 1.2 × height. Valve length 6 × width, base widest in genus, segment distal to clasper thin, straight; cucullus, very weak, rounded; sacculus length 0.5 × and width 0.8 × valve; clasper thick, expanded distal part 1 × valve width, right larger than left, mesial surface spinulose, dorsum lacking process other than ampulla, ventral process long, curved slightly dorsad, claw shaped; ampulla base at dorsal valve ⅔ from base to apex, base broad, perpendicular to valve, curved 180° distad; digitus weak. Phallus and vesica as in genus description; vesica diverticulum slightly smaller than in the other species.

*Female genitalia* (Figs 12, 15): Posterolateral sternite A7 bisected by transverse sulcus, deepest laterally; posterior margin strongly concave, nearly semicircular. Papilla analis, segment A8, and bursa copulatrix as in genus description; ostium bursae leathery without median cleft.

##### Etymology.

I am pleased to name this moth for Merrill Peterson of Bellingham, Washington in recognition of his work on the Pacific Northwest Moths website (http://pnwmoths.biol.wwu.edu). This site is a well-illustrated interactive guide to the moths of the northwestern United States and British Columbia, Canada.

##### Distribution and ecology.

*Rhabdorthodespetersoni* is known only from the type locality at 3150 meters on Cerro Potosi, the highest peak in the Sierra Madre Oriental, Mexico (Figure 16). The habitat is unknown, although it is most likely forest. Both known specimens were collected in mid-July. The early stages are unknown.

##### Remarks.

This species was found by J. Donald Lafontaine amongst unsorted Mexican material at the CNC. Although this moth is only known from Mexico it could conceivably occur in western Texas, particularly in the Davis and Chisos mountains. These ranges are the northernmost extension of the Sierra Madre Oriental.

## Supplementary Material

XML Treatment for Rhabdorthodes

XML Treatment for Rhabdorthodes pattersoni

XML Treatment for Rhabdorthodes durango

XML Treatment for Rhabdorthodes petersoni
